# Automatic generation of alloreactivity-reduced donor lymphocytes and hematopoietic stem cells from the same mobilized apheresis product

**DOI:** 10.1186/s12967-023-04738-8

**Published:** 2023-11-25

**Authors:** E. Wiercinska, P. Quade-Lyssy, C. Hümmer, J. Beifuß, K. Akarkach, C. Poppe, V. Olevska, J. Dzionek, H. Lahnor, A. Bosio, E. Papanikolaou, Halvard Bonig

**Affiliations:** 1 Department of Cellular Therapeutics (GMP), German Red Cross Blood Service BaWü-He, Institute Frankfurt, Frankfurt, Germany; 2 Miltenyi Biotec B.V. & CO. KG, Bergisch Gladbach, Germany; 3 Miltenyi Biomedicine GmbH, Bergisch Gladbach, Germany; 4 Laboratory of Biology, School of Medicine, National and Kapodistrian University of Athens, Athens, Greece; 5 Institute for Transfusion Medicine and Immunohematology, Goethe University, Frankfurt, Germany; 6 Department of Medicine, Division of Hematology, University of Washington, Seattle, WA USA; 7 DRK-BSD BaWüHe, Sandhofstraße 1, 60528 Frankfurt, Germany

**Keywords:** Haploidentical HSCT, TCRαβ/CD19-depleted graft, CD45RA-depleted DLI, GMP-compatible process, CliniMACS Prodigy

## Abstract

**Introduction:**

In vitro or in vivo depletion of alloreactive T cells can facilitate haplo-identical hematopoietic stem cell transplantation (HSCT). Very satisfactory transplant outcomes were thus reported for TCRαβ/CD19-depleted hematopoietic stem/progenitor cell (HSPC) grafts. The current semi-automatic manufacturing process on the CliniMACS Plus, although robust, still requires a significant amount of manual labor to be completed. Towards advancing and further facilitating large scale cell processing, a new TCRαβ/CD19 depletion module combined with the previously described CD45RA depletion module (to serve as allo-reactivity attenuated donor lymphocyte infusion) was established on the CliniMACS Prodigy.

**Methods:**

We evaluated six apheresis products from G-CSF-mobilized volunteer donors which were split automatically by the Prodigy, one portion each depleted of CD45RA^+^ or of TCRαβ^+^ and CD19^+^ cells. We investigated critical quality attributes for both products. Products were assessed for recovery of HSPCs and mature subsets, as well as depletion efficiency of targeted cells using flow cytometry. Effects of apheresis and product age post 48 h storage at 2–6 °C as well as freeze-thawing on product viability and recovery of WBC and HPSCs were assessed by flow cytometry.

**Results:**

Ten sequential automatic processes were completed with minimal hands-on time beyond tubing set installation. Depletion efficiency of CD45RA^+^ resp. TCRαβ^+^ and CD19^+^ cells was equivalent to previous reports, achieving mean depletions of 4 log of targeted cells for both products. HSPC products retained TCRγδ^+^ and NK cells. 48 h storage of apheresis product was associated with the expected modest loss of HSPCs, but depletions remained efficient. Depleted products were stable until at least 72 h after apheresis with stem cell viabilities > 90%. Freeze-thawing resulted in loss of NK cells; post-thaw recovery of viable CD45^+^ and HSPCs was > 70% and in line with expectation.

**Conclusion:**

The closed, GMP-compatible process generates two separate medicinal products from the same mobilized apheresis product. The CD45RA-depleted products contained functional memory T cells, whereas the TCRαβ/CD19-depleted products included HSPCs, TCRγδ^+^ and NK cells. Both products are predicted to be effectively depleted of GVH-reactivity while providing immunological surveillance, in support of haplo-identical HSCT.

**Graphical Abstract:**

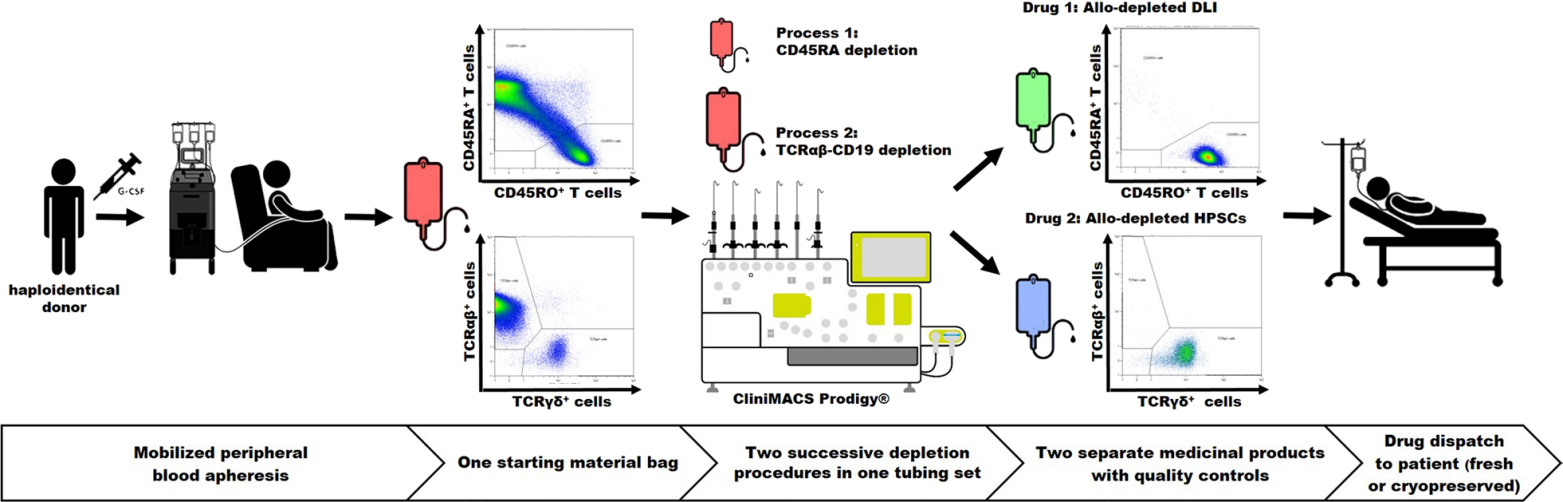

**Supplementary Information:**

The online version contains supplementary material available at 10.1186/s12967-023-04738-8.

## Introduction

In the context of allogeneic HSCT, T lymphocytes are at the same time contaminant, causing potentially lethal graft-versus-host disease (GVHD), and active component, controlling residual blasts (graft-versus-leukemia, GVL) as well as opportunistic (viral) infections. The tolerable dose of T cells varies widely, HLA match being its strongest predictor. In matched-donor HSCT, despite an approximately 40-fold excess of T cells over HSPCs in a G-CSF-mobilized peripheral blood HSPC graft, immunosuppressive prophylaxis typically suffices to establish a balance between desired and noxious effects of T cells. In haplo-identical HSCT, however, strong T cell depletion is required in order to manage allo-reactivity. The classical T cell depleted graft, purified CD34^+^ cells, if carefully manufactured, can be administered even in an HLA haplo-identical constellation without need for prophylactic immunosuppression, and i.e., is safe with respect to GVHD. However, delayed immune reconstitution poses the risks of relapse and infection with opportunistic agents due to lack of immunological surveillance of residual blasts and missing adaptive immunity associated with high overall transplant-related-mortality of around 40% [1–3]. CD3/CD19-depleted HSPC products had previously been tried, but despite clinically satisfactory outcomes, difficulties in consistently generating sufficiently T cell-poor grafts had hampered its general clinical breakthrough [4]. The quest, therefore, for processes yielding allo-reactivity-reduced, yet immunologically reasonably competent grafts has continued, recently proposing T cell receptor αβ T cell and B cell-depleted (TCRαβ/CD19-depleted) HSPC products for mismatched allo-HSCT as clinically relevantly might be superior to CD34-selected and CD3/CD19-depleted grafts [5]. TCRγδ^+^ T cells were, in the meantime, identified as low-risk for causing GVHD and possibly even beneficial [6, 7], so that depletion of only TCRαβ^+^ instead of all T cells (all CD3^+^ cells) was developed as an alternative GVHD-free strategy. Sequential labelling of TCRαβ^+^ cells with biotinylated antibody and microbeads conjugated to anti-biotin have been proven efficient in loading sufficient numbers of paramagnetic particles onto the cells for consistently effective depletion. CD19^+^ cells are typically co-depleted to diminish the risk of post-transplant lymphoproliferative disease (PTLD), since CD19^+^ cells can serve as a reservoir for latent Epstein-Barr virus. EBV seroprevalence already in young adults exceeding 95% [5, 8], we propose to include B cell depletion for all donors instead of validating a second process without CD19 depletion for the rare EBV-naïve donor. The process of combined TCRαβ and CD19 depletion as HSPC graft manipulation was previously reported by us and others [9] and showed robust and clinically useful resulting products. However, the existing processes on the CliniMACS Plus were laborious, requiring long hands-on process times in the GMP clean room, followed by quality control testing until late in the evening. Despite apparent advantages, therefore, TCRαβ/CD19-depleted grafts have thus far not conquered the haplo-transplant market. The other reason might be the widely used option of haplo-HSCT with PT/Cy which is much cheaper and easy to handle without the need to manipulate the haplo-identical graft. In 2015, the first application for TCRαβ^+^ and CD19^+^ depletion was launched for the CliniMACS Prodigy (Miltenyi Biotec) in an automated and closed system [10]. Despite some issues regarding the software version and the need to add extra waste bags and to transfer the final product into infusion bags, Haastrup et al. [10] reported the first ten automated TCRαβ/CD19 depletion procedures from haplo-identical donors for clinical use that were successfully performed on the CliniMACS Prodigy.

Because naïve T cells are highly variable in their TCR repertoire, they have been implicated in GVHD. Naïve T cells are expressing the RA splice variant of CD45. CD45RA depletion from the graft was thus proposed as a strategy to generate donor lymphocyte infusion (DLI) products containing potentially GVL reactivity and anti-infectious immunity but with minimal risk of causing GVHD [11–14]. As expression of CD45RA is not restricted to T cells, but is also found on approximately one fourth of HSPCs [15–17], CD45RA depletion is not suited for HSPC graft manufacturing. As we previously reported [15], CD45RA-depleted DLI can be automatically manufactured with CliniMACS Prodigy (Miltenyi Biotec, Bergisch Gladbach, Germany). A number of clinical reports indeed seem to support GVHD safety and GVL efficacy of modest doses of CD45RA-depleted DLIs in the context of matched-donor or haplo-identical transplantations [18–21]. We therefore sought to utilize a cell separation strategy comprised of sequential depletion of CD45RA cells from a small aliquot of mobilized apheresis product to serve as DLI, followed by TCRαβ/CD19 depletion of the rest of the product to be used as HSPC graft, on the automatic cell manufacturing device CliniMACS Prodigy. The automated process would start as early as possible after the end of the apheresis and proceed overnight unattended, so that the CD45RA-depleted DLI would be ready two hours after process start and the TCRαβ/CD19-depleted HSPC graft in the morning of the subsequent day. At that point, quality controls can be performed, the allo-reactivity depleted DLI is cryopreserved for later adaptive transfer, the HSPC product labeled, released, and dispatched to the transplant center. As our results show, the new process is scalable, robust and highly efficient. Products generated in this way are predicted to provide similar transplant outcomes as previously reported for manually manipulated grafts [9]. Unless the manual process is also performed overnight, CliniMACS Prodigy products will be half a day less old than CliniMACS Plus products at the time of cryopreservation or release, which may be an additional benefit. Conceptually, this effort illustrates the ability of automated platforms like CliniMACS Prodigy to accommodate cell processing protocols with increasingly more challenging schedules.

## Materials and methods

### Starting material

Starting material were G-CSF-mobilized leukapheresis products from consenting volunteer donors who were undergoing stem cell apheresis as matched unrelated donors and whose good mobilization and favorable donor to recipient weight ratio allowed for additional cell collection (“research apheresis”) within the 300 min’ duration of a leukapheresis session. Performance of such research aphereses, written informed consent provided, was approved by the Ethics Committee of Goethe University Medical School, Frankfurt, Germany (approval number 286/13). Aphereses were performed with Spectra Optia cMNC (Terumo BCT, Lakewood, CO), as described [22]. A total of six apheresis products were collected, for a total of ten depletion runs.

### Cell processing

**Fig. 1:**
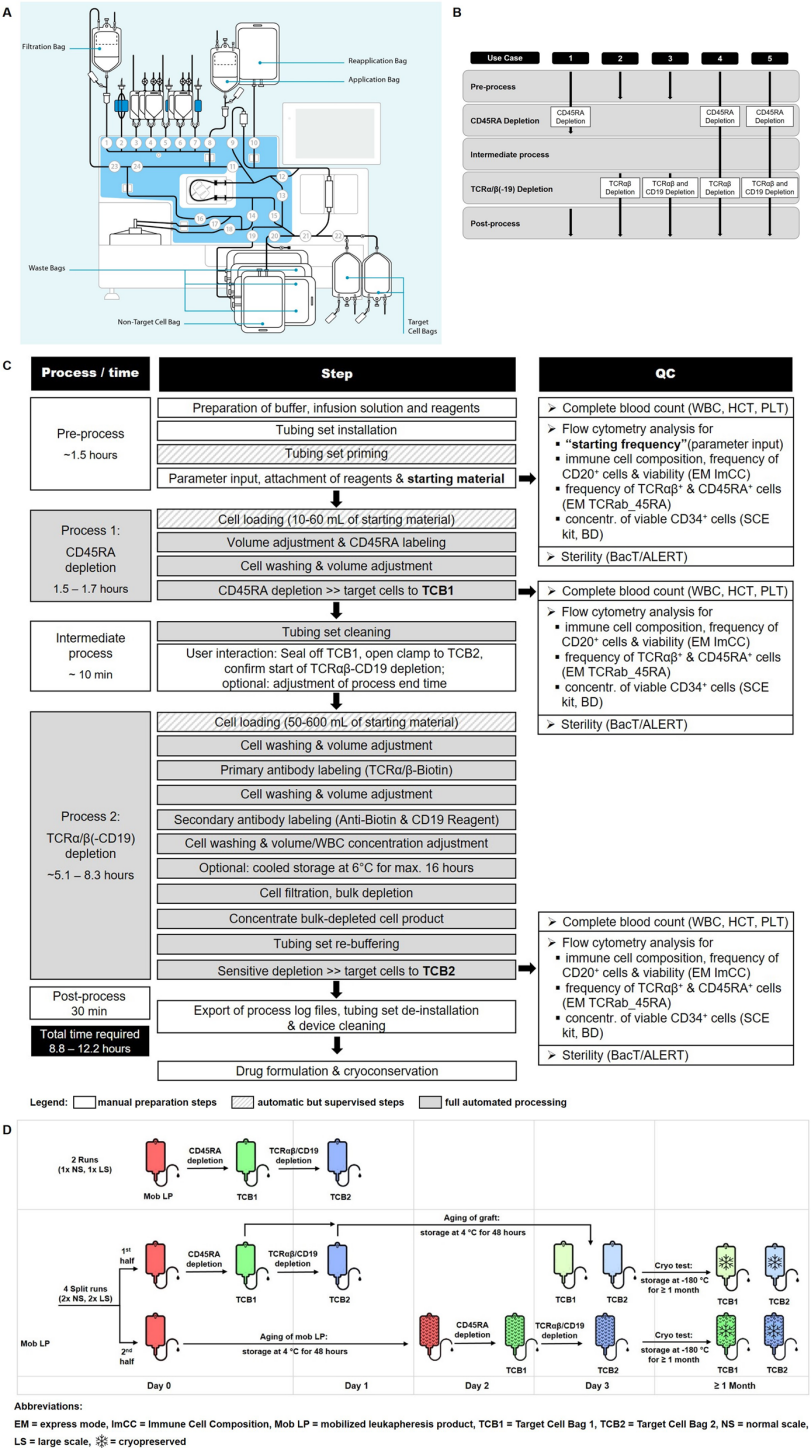
Overview of the study approach. **A** CliniMACS Prodigy TS 320 setup for the automated LP-TCRαβ-19-45RA Depletion procedure. A brief description of the components and preparation steps of the tubing set is added in Additional file 1: Figure S1. Starting material (mobilized apheresis products, mob LP) is automatically split for CD45RA and for TCRαβ/CD19 depletion which are performed sequentially and which results in two separate cell products. Target Cell Bag 1 (TCB1) contains a cell product depleted of naïve T cells and other CD45RA+ leukocyte subsets. Target Cell Bag 2 (TCB2) includes TCRγδ+ cells, HSPCs, NK cells, among others. Copyright © 2023 Miltenyi Biotec B.V. & Co. KG. All rights reserved. **B** Diagram showing the different use cases of the depletion software. **C** Workflow diagram of the manufacturing procedure with timeline and quality controls. White, dashed and gray shading mark hands-on, automatic-supervised and autonomous preparation steps, respectively. **D** Graphical description and schedule of experimental setup. All processes consisted of sequential CD45RA and TCRαβ/CD19 depletion. Ten depletion procedures from six mob LP (red) were performed. Four thereof were split runs to compare immediate processing vs. processing after 48 h storage at 2–6 °C to analyze the effect of apheresis product aging (mob LP, patterned red) on manufacturing performance and product quality (TCB1, patterned green and TCB2, patterned blue, respectively). Additionally, the effect of aging on the depleted products (TCB1, light green and TCB2, light blue, respectively) was assessed after storage at 2–6 °C for 48 h. Normal scale (5/10 runs NS) and large scale (5/10 runs LS) depletion reagents were used to test both, for depletion performance and final product quality. Final products from four split runs were cryopreserved to test how freeze–thaw procedure affects the final product quality at the end of the of their shelf life (TCB1, light green with snowflake and TCB2, light blue with snowflake, respectively, as well as TCB1, patterned green with snowflake and TCB2, patterned blue with snowflake, resepctively)

Four of the six products were split for immediate processing vs. processing after 48 h, to test how apheresis product age affects performance of the depletion and final product quality. Of the 10 depletion processes, five each were performed with standard (normal scale, NS) or XL (large scale, LS) reagents and protocols, to test the performance of both process scales. All separations were performed with the CliniMACS Prodigy cell processing device (previously introduced; [15, 23–25]) and version 1.0.3 of the depletion software application LP-TCRab-19-45RA Depletion (Fig. 1A). As shown in Fig. 1B, the software allows for different use cases, namely: Use case 1 only for CD45RA depletion, use case 2 only for TCRαβ depletion, use case 3 for combined TCRαβ and CD19 depletion, use case 4 for combined CD45RA and TCRαβ depletion and finally use case 5 for combined CD45RA, TCRαβ and CD19 depletion. All our processes consisted of sequential CD45RA and TCRαβ/CD19 depletion (use case 5). A detailed workflow of the process used in this manuscript is shown in Fig. 1C. Both target products from four depletion runs were cryopreserved after storage at 2–6 °C for 48 h at a WBC concentration not exceeding 200 × 10^6^ cells/mL in 5% human serum albumin (HSA; CSL Behring, Marburg, Germany) in normal saline supplemented with 7.5% (v/v final) dimethyl sulfoxide (DMSO; WAK Chemie, Steinbach/Ts., Germany) in ethylene vinyl acetate (EVA) CryoMACS Freezing Bags (Miltenyi Biotec), using controlled-rate freezers with an initial − 1 K/min ramp and rapid cooling at the eutectic point, as described [4].

### Depletion reagents and method

Selection was performed with one vial each of CliniMACS TCRαβ-Biotin XL, Anti-Biotin Reagent XL and CliniMACS CD19 Reagent XL (large-scale process) or one vial CliniMACS TCRαβ-Biotin and two vials each for CliniMACS Anti-Biotin Reagent and CD19 Reagent (normal scale process) for the TCRαβ/CD19 depletion, one vial of CD45RA Reagent XS for the CD45RA depletion, three 3-Liter bags of CliniMACS PBS/EDTA Buffer supplemented with 20% w/v medical grade human serum albumin (HSA, CSL Behring, Marburg, Germany) to a final concentration of 0.5% w/v HSA as process buffer, two 1-Liter bags of isotonic sodium chloride (B. Braun, Melsungen, Germany) supplemented with 20% w/v medical grade HSA to a final concentration of 0.5% w/v HSA as infusion solution for injection use, one 1-Liter bag of aqua bidest. (Ampuwa, Fresenius Kabi, Bad Homburg, Germany) as rinsing solution, a CliniMACS Prodigy Tubing Set TS 320, the CliniMACS Prodigy cell processing device (previously introduced; [15, 23–25]) and version 1.0.3 of the depletion application software LP-TCRab-19-45RA Depletion. Briefly, after installing and priming the tubing set TS 320, transferring the labelling reagents into the designated reagent bags, entering the separation-related cell product information and tube-welding connection of the starting material—for CD45RA an aliquot of approximately 20 mL containing 4–9 × 10^8^ total WBCs (process capacity is between 10 and 60 mL containing up to 2.4 × 10^9^ CD45RA^+^ cells from 6 × 10^9^ total WBCs) was taken automatically by the process from the apheresis product—the CD45RA depletion process was started and proceeded fully automatically, as described [15]. The CD45RA-depleted target product was provided in NaCl-HSA solution and collected in Target Cell Bag 1 (TCB1), in our case ~ 120 mL. After automatic rinsing of the tubing set, the TCRαβ/CD19 depletion proceeded subsequently with the residual starting material. A desired process end time was entered, to allow overnight processing and provision of the depleted cell product in the next morning. The process capacity is between 50 and 600 mL of starting cell product containing up to 24 × 10^9^ TCRαβ^+^ cells and 10 × 10^9^ CD19^+^ cells from 60 × 10^9^ total WBCs or up to 48 × 10^9^ TCRαβ^+^ cells and 15 × 10^9^ CD19^+^ cells from 120 × 10^9^ total WBCs, for normal and large-scale processing, respectively. The TCRαβ/CD19-depleted target product was provided in approximately 250 mL of NaCl-HSA solution and collected in Target Cell Bag 2 (TCB2). A summary table regarding relevant parameters (e.g. cell input, volume) and total cell numbers for relevant cell populations for all ten depletion processes was added as Additional file 1: Table S1. All reagents and consumables used were obtained from Miltenyi Biotec, Bergisch Gladbach, Germany, unless otherwise noted. An overview of the experimental workflow is depicted also in Fig. 1D.

### Flow cytometric analyses

**Fig. 2:**
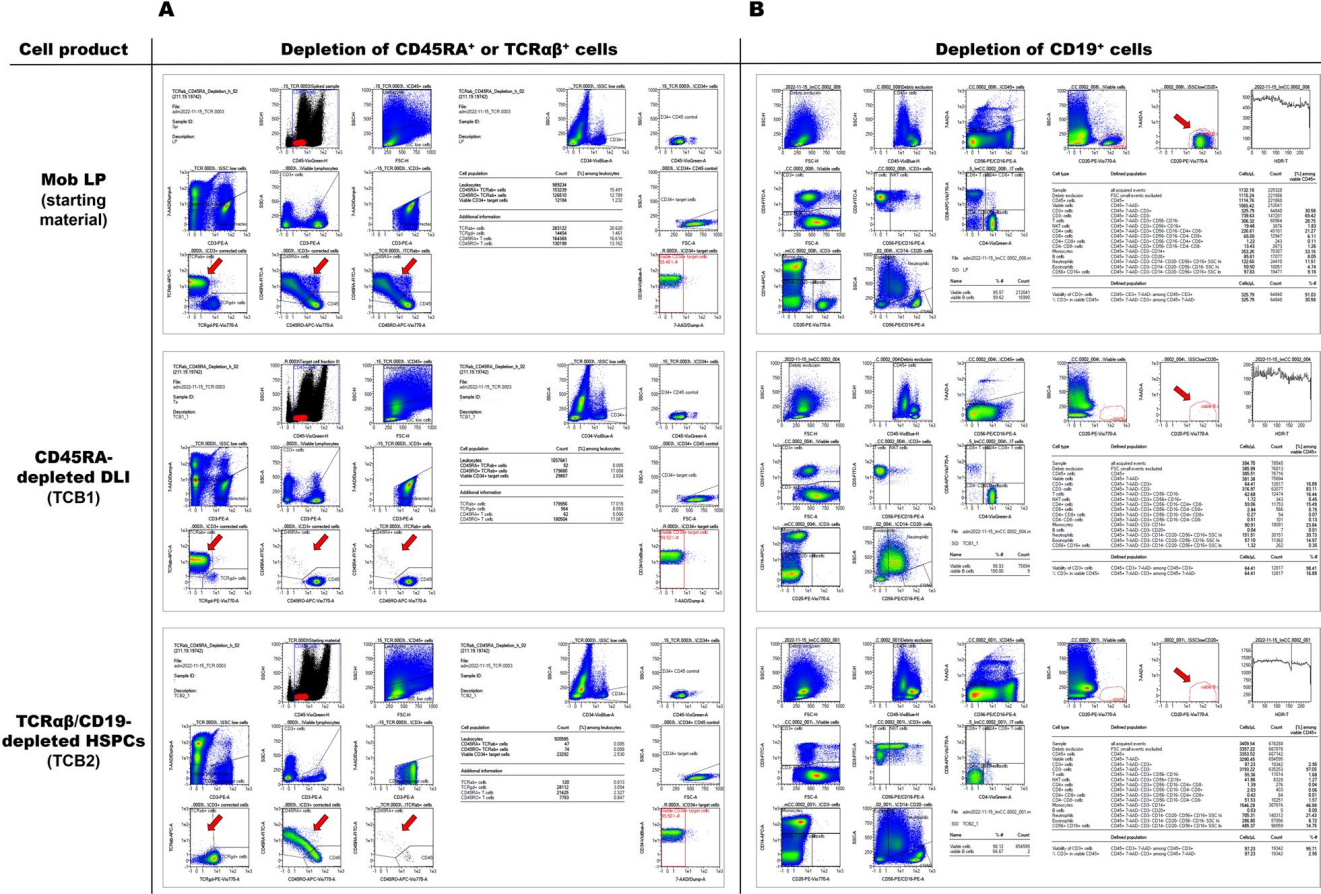
Flow cytometric panels for characterization of CD45RA-depleted DLI (TCB1) and TCRαβ/CD19-depleted HSPCs (TCB2) in comparison to apheresis product (mob LP). **A** The gating strategy using the express mode “TCRab_CD45RA_Depletion_h_02” determines automatically the TCRαβ^+^ and CD45RA^+^ frequencies in the different fractions before and after depletion. Additionally, this panel includes the identification of the CD34^+^ cell population among CD45^+^ leukocytes, however the stem cell enumeration was determined using the BD’s CE-certified SCE kit following stringent ISHAGE gating (panel not shown, [26]). The arrows indicate, left to right, TCRαβ over TCRγδ gated on T cells, CD45RA over CD45RO gated on T cells, and CD45RA over CD45RO gated on TCRαβ T cells. The same analysis is shown, from top to bottom, for apheresis product (Mob LP), CD45RA-depleted DLI and TCRαβ/CD19-depleted HSPCs. CD45RA-depletion depletes almost all TCRαβ T cells, but the inverse is not true for TCRαβ-depletion: the lower panel demonstrates a high frequency of CD45RA^+^ T cells (lower panel, middle) despite almost complete depletion of TCRαβ T cells (lower panel, left). **B** The gating strategy using the express mode “Immune_Cell_Composition_human” facilitates automatic determination of cell concentration, viability of leukocytes, cell composition and frequency of B cells. The arrows identify B cells. Both CD45RA-depletion (middle panel) and TCRαβ/CD19-depletion (lower panel) almost completely deplete B cells from DLI (middle) or HSPC product (lower)

Flow cytometric analyses were performed with the MACSQuant flow cytometer equipped with the standard three-laser set-up and filters using the MACSQuantify software, version 2.11. We validated two automated express mode analysis algorithms developed by Miltenyi Biotec for the application described here, “TCRab-CD45RA_depletion_h_02” and “Immune_Cell_Composition_human” for assessment of the cell frequencies of the depleted cell populations and determination of the residual immune cell repertoire, respectively. Representative examples for each of the panels and the gating strategy are given in Fig. 2. Sample aliquots of starting material before (mobilized leukapheresis, mob LP) and after processing (target cell bag 1 & 2, TCB1 & TCB2), at the end of the shelf life (after 48 h of storage) and post-thaw were investigated. To assess CD45RA resp. TCRαβ and CD19 depletion efficiency, 2 × 10^6^ cells of the starting cell product (mob LP), non-target cells (NTCB) or the depleted target cells (TCB1 and TCB2, respectively) were stained with antibodies against CD3 (PE, clone REA613), CD45 (VioGreen, clone REA747), CD45RO (APC-Vio 770, clone REA611), CD45RA (FITC, REA1047), TCRαβ (APC, clone BW242/412), TCRγδ (PE-Vio 770, clone 11F2), CD34 (VioBlue, clone AC136), CD14 (PerCP-Vio 770, clone REA599), CD15 (PerCP-Vio770, clone VIMC6) and incubated for 10 min at 2–6 °C with 20 µL FcR-Blocking Reagent and filled up with buffer to 100 µL. After incubation and red blood cell lysis, cells were resuspended in 500 µL CliniMACS PBS/EDTA Buffer supplemented with 0.5% w/v HSA. After automatic labeling with 7-AAD, 450 µL (TCB1 and TCB2) or 200 µL (mob LP or NTCB) sample volume were acquired immediately. The immune cell composition panel was as follows: CD45 (VioBlue, clone REA747), CD3 (FITC, clone: REA613), CD8 (APC-Vio 770, clone REA734), CD4 (VioGreen, clone VIT4), CD14 (APC, clone REA599), CD20 (PE-Vio 770, clone REA780), CD16 (PE, clone REA423) and CD56 (PE, clone REA196). 100 µL cell suspension, WBC concentration should be between 1 × 10^6^ and 1 × 10^8^ cells/mL, was stained with 100 µL staining cocktail containing 7-AAD for 10 min at 2–6 °C. After blood cell lysis in 1800 µL lysis buffer for 10 min in the dark at room temperature, samples were acquired immediately without washing. Uptake volume was set to 200 µL. Examples of the acquisition and analysis worksheet are given as Fig. 2. “Stem cells” were enumerated with the SCE stem cell enumeration kit (Becton–Dickinson, Heidelberg, Germany) and strict ISHAGE gating, as described previously [26].

### Colony-forming unit-culture assay

To confirm preservation of clonogenic activity of the stem cell graft after processing and especially after freeze-thawing at different time-points, aliquots containing an equivalent of 1000 7-AAD^- ^excluding CD34^+^ cells/2 mL of myeloid outgrowth-supporting cytokine-replete semi-solid culture media (StemMACS HSC-CFU lite with Epo, human) were plated into two 35 mm cell culture dishes. After 12–14 days, myeloid colonies were enumerated.

### Statistics

Descriptive statistics were calculated in GraphPad Prism 5 (version 5.01). All data were tested for statistical significance using Student’s t-test with Bonferroni correction for multiple testing where appropriate. Shown are individual runs and mean values ± SEM, n = 4–10 individual runs; ns (not significant) indicates a p > 0.05, *: p < 0.05, **: p < 0.01, ***: p < 0.001. p-values are reported above the corresponding groups in the graphs.

## Results

### Depletion process

We validated “use case 5” of the novel automated CliniMACS Prodigy LP-TCRab-19-45RA Depletion application which consists of sequential CD45RA- and TCRαβ/CD19 depletion (Fig. 1B). The entire procedure was performed in a single tubing set with one GMP operator present. Total and labelled cell numbers in the starting material determine whether reagents for normal scale or large scale should be used. Hands-on time before starting the automatic depletion process was 90 min for buffer preparation, instrument set-up, transfer of reagents, and supervised operation during instrument priming and cell loading. A brief user interaction was needed during the intermediate process for sealing off TCB1, opening the clamp to TCB2, adjusting the process end time (optional) and finally confirming the start of TCRαβ/CD19 depletion. After completion of the automated process, tubing set de-installation, instrument cleaning, and export of process log files took 30 min. The entire process required a process time of 8.8 to 12.2 h depending on cell numbers (Fig. 1C). Combined hands-on time for documentation, kit mounting and dismounting as well as cleaning was 2.5 operator hours. Unsupervised process operation lasts a minimum of 1.5–1.7 h for a CD45RA depletion, 5.1–8.3 h for a TCRαβ/CD19 depletion, and approximately 7 to 10 h for both processes to complete. For staff convenience the process end time was adjusted to 8 a.m., i.e., for an actual process time of approximately 14 h, automatically pausing the process after labeling and removal of reagent excess in order to reach the desired end time. Both depletion processes proceeded without interruption and were able to efficiently deplete CD45RA^+^ and TCRαβ^+^/CD19^+^ cells in all ten validation runs. Sterility was confirmed for all products using the BacT/ALERT 3D system (bioMérieux, Nürtingen, Germany).

### CD45RA depletion efficiency

**Fig. 3:**
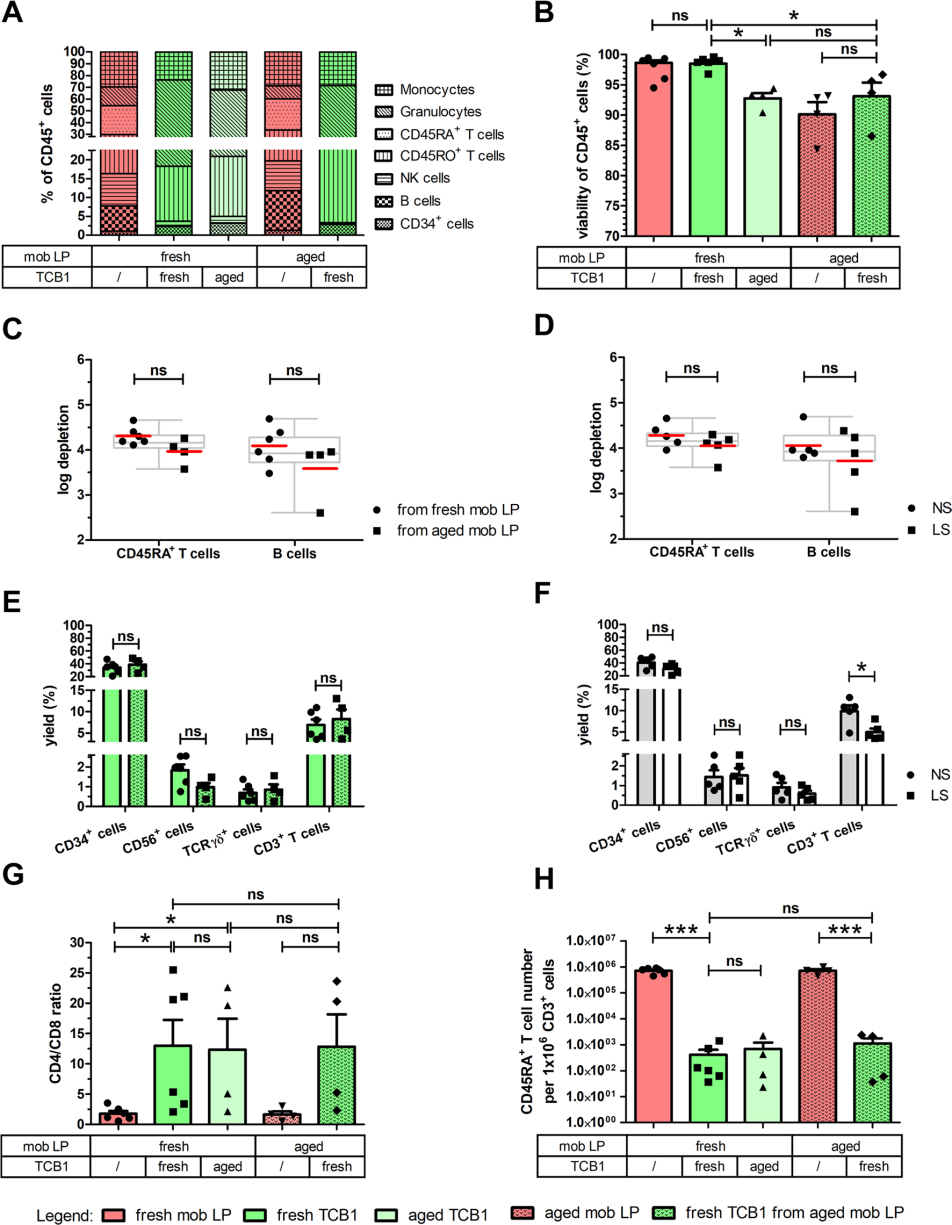
Characterization of CD45RA-depleted DLIs. Apheresis product was either processed immediately after apheresis (fresh mob LP, red) or after storage at 2–6 °C for 48 h (aged mob LP, patterned red). CD45RA-depleted DLI product (fresh TCB1, green) was analyzed immediately after processing of fresh or aged mob LP or after storage at 2–6 °C for 48 h (aged TCB1, light green). **A** Relative contribution of individual mature leukocyte subpopulations in starting material and target cell fractions among CD45^+^ leukocytes at the different time-points. The percentage of each subtype was normalized to 100% CD45^+^ leukocytes. **B** Percentage of viable CD45^+^7-AAD^−^ leukocytes in all fractions at different time-points. Depletion efficiency of naïve CD45RA^+^ T cells and B cells (universally CD45RA^+^) was compared for products (**C**) derived from mobilized apheresis product (mob LP), processed immediately after apheresis (fresh mob LP) or after storage at 2–6 °C for 48 h (aged mob LP) and **D** derived from processes using NS or LS depletion reagents, respectively. Log depletion for each individual run is depicted with dots and squares representing products generated from fresh and aged mob LP or from NS and LS reagents, respectively. Red line indicates mean values achieving mean log depletions of around 4 log in all tested settings. Box plots and whiskers represent median with interquartile range and min and max values of all 10 depletion runs, respectively. Mean yield of CD34^+^ stem cells, NK cells, TCRγδ^+^ cells and CD3^+^ T cells among total CD45^+^ cells in depleted products (**E**) derived from fresh or aged mob LP and **F** from processes using NS or LS depletion reagents showing a high loss of cells of interest. **G** The CD4/CD8 ratio among total T cells was 6.5-fold increased in all depleted products. **H** Residual alloreactive CD45RA^+^ T cells in CD45RA-depleted products were calculated per 1 × 10^6^ CD3^+^ T cells which are the main active component of a DLI product. All data were tested for statistical significance using Student’s t-test. Shown is mean ± SEM, n = 4–10 individual runs; ns (not significant) indicates a p > 0.05, *: p < 0.05, ***: p < 0.001. P-values are reported above the corresponding groups

A continuous population ranging from isolated CD45RA to isolated CD45RO expression and a large double-positive population in between characterizes the distribution of the CD45 isoforms on T cells, as shown in Fig. 2A. Although reliable gating on CD45RA-negative T cells might be challenging in the starting material, a distinct CD45RA-negative T cell population is left after depletion of CD45RA^+^ cells in TCB1. Mean depletion of CD45RA^+^ cells according to flow cytometry was from a starting frequency in excess of 25% to nearly complete (0.01 ± 0.00%, mean ± SEM) (Fig. 3A) resulting in mean depletions of 4 log regardless of age of starting material (fresh vs. aged) or reagent scale used (NS vs. LS) (Fig. 3C, & D). Similar mean log depletions were achieved for B cells which are universally CD45RA-positive (Fig. 3C & D). The percentage of viable CD45^+^7-AAD^−^ leukocytes in all fractions at different time-points met our defined internal specification criteria of ≥ 70%, showing mean viabilities over ≥ 90% (Fig. 3B).

### Immunological differentials of CD45RA-depleted products

The relative frequency of granulocytes in TCB1 compared to the starting material was increased by 3.5-fold, due to the almost complete removal of NK cells and B cells and significant depletion of naïve T cells by CD45RA depletion (Fig. 3A, E, F). Besides CD45RO^+^ T cells and granulocytes, the final product contained monocytes at a frequency approximately equivalent to that in the starting population and, despite removal of the CD45RA-expressing CD34^+^ cells, an almost two-fold increased mean frequency of CD34^+^ cells (2.32% vs. 1.13% in the starting material) was observed. However, total cell numbers were reduced by 5.8-fold for CD45^+^ cells and by 2.7-fold for CD34^+^ cells (data not shown) resulting in mean yields of CD34^+^ cells of only about 40% and modest mean yields of CD3^+^ T cells in order of magnitude of 5–10% (Fig. 3E & F). Mean yields of the potential killer effector CD56^+^ cells and TCRγδ^+^ cells were around 1–2% (Fig. 3E & F).

### T cell subset analyses

As expected, naïve and effector T cells were almost completely removed by CD45RA depletion; the frequency of CD8^+^ T cells was reduced from 35% (range 22–56%) in starting material to 13% (range 4–28%) in TCB1. At the same time, the frequency of CD4^+^ cells among total T cells in TCB1 was increased to > 80% of all T cells, skewing the mean CD4/CD8 ratio from 2:1 to 13:1 (Fig. 3G). The occurrence of GVHD, being the main limiting toxicity of DLI, depends on the alloreactive T cell dose. Figure 3H depicts the residual total cell number of potentially alloreactive CD45RA^+^ T cells calculated per 1 × 10^6^ CD3^+^ T cells. A safe, yet effective dose of CD45RA^−^ T cells has not yet been established, though.

### TCRαβ/CD19 depletion efficiency

**Fig. 4:**
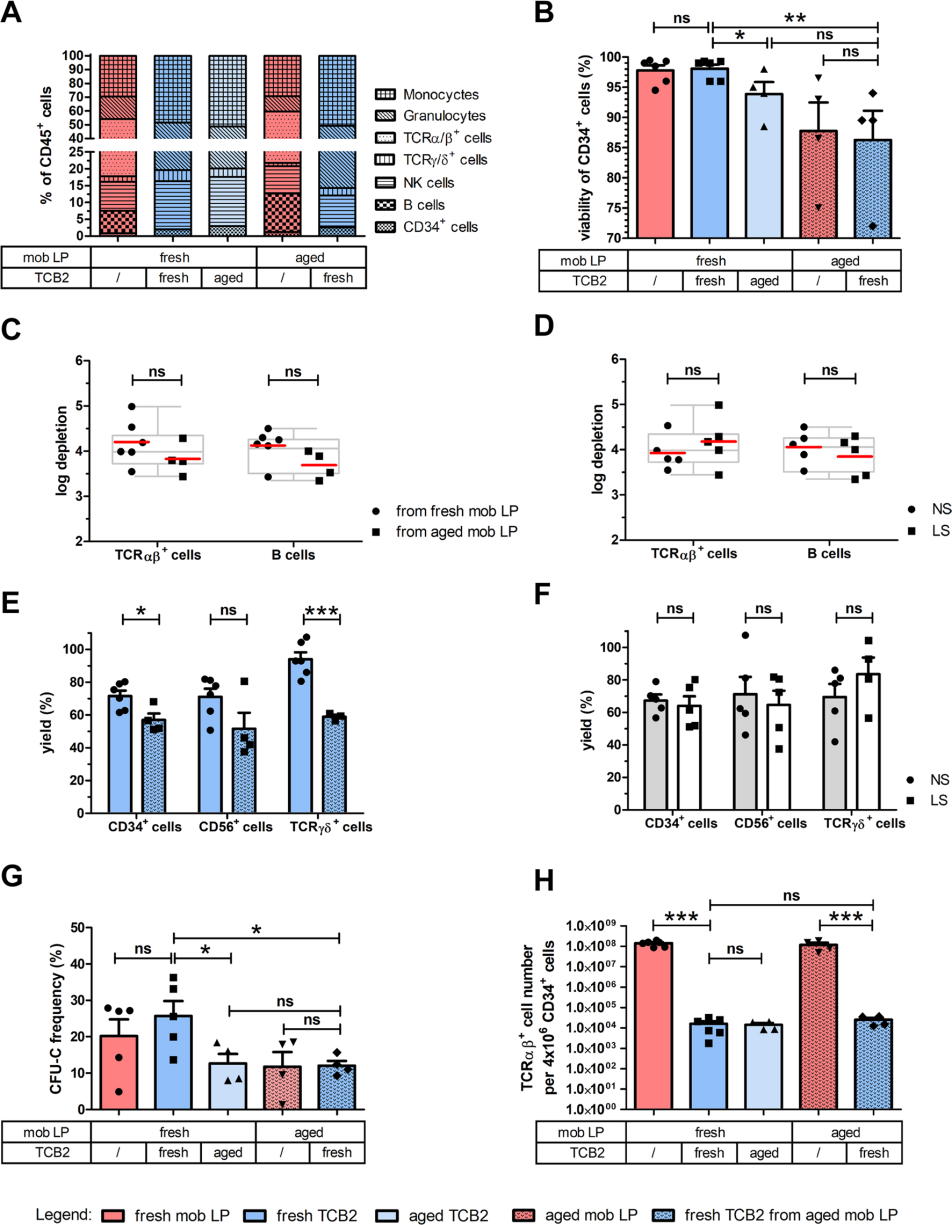
Characterization of TCRαβ/CD19-depleted HSPC grafts. Apheresis product was either processed immediately after apheresis (fresh mob LP, red) or after storage at 2–6 °C for 48 h (aged mob LP, patterned red). The TCRαβ/CD19-depleted product (fresh TCB2, blue) was determined immediately after processing of fresh or aged mob LP or after storage at 2–6 °C for 48 h (aged TCB2, light blue). **A** Relative contribution of individual mature leukocyte subpopulations in starting material and target cell fractions among CD45^+^ leukocytes at the different time-points. The percentage of each subtype was normalized to 100% CD45^+^ leukocytes. **B** Percentage of viable CD45^+^7-AAD^−^ leukocytes in all fractions at different time-points. The efficiency of depletion of TCRαβ^+^ T cells and CD19^+^ B cells was compared for products **C** derived from mobilized apheresis product (mob LP) processed immediately after apheresis (fresh mob LP) or after storage at 2–6 °C for 48 h (aged mob LP) and **D** derived from processes using NS or LS depletion reagents, respectively. Log depletion for each individual run is depicted with dots and squares representing products generated from fresh and aged mob LP or from NS and LS reagents, respectively. Red line indicates mean values achieving mean depletion efficiency of around 4 log in all tested settings. Box plots and whiskers represent median with interquartile range and min and max values of all 10 depletion runs, respectively. Mean yield of CD34^+^ stem cells, NK cells and TCRγδ^+^ cells among total CD45^+^ cells in depleted products (**E**) derived from fresh or aged mob LP and **F** from processes using NS or LS depletion reagents showing a good recovery of cells of interest. **G** A CFU-C assay was performed via a 14-day culture followed by counting total number of colonies of mob LP and TCB2 at different time-points. Each individual run is plotted, as is mean ± SEM. **H** The residual alloreactive TCRαβ^+^ T cell number in HSPC graft was calculated per 4 × 10^6^ CD34^+^ stem cells representing the transplant dosage of 4 × 10^6^ CD34^+^ stem cells per kg as the main active component. All data were tested for statistical significance of differences using Student’s t-test. Shown are individual runs and mean ± SEM, n = 4–10 individual runs; ns (not significant) indicates a p > 0.05, *: p < 0.05, **: p < 0.01, ***: p < 0.001. P-values are reported above the corresponding groups

The immune cell composition of the TCRαβ/CD19- depleted product (TCB2) was not systematically different in freshly processed apheresis vs. aged products (Fig. 4A). Monocytes, granulocytes and even CD34^+^ and NK cells were passively enriched due to nearly complete depletion of the highly abundant TCRαβ^+^ T cells and B cells. Mean depletion efficiency for TCRαβ^+^ and CD19^+^ cells exceeded 4 log levels regardless of apheresis product age or process scale (Fig. 4C & D). HSPCs were recovered almost quantitatively (70%, 62–80%; mean, range; Fig. 4E & F) with very good viability > 90% (Fig. 4B). Clonogenic assays confirmed functionality of TCRαβ/CD19-depleted HSPC products, although colony formation was significantly reduced from aged products and from products generated from aged leukapheresis material (Fig. 4G). To ensure reliable engraftment in the allogeneic setting, the targeted HSPC dose for transplantation of mobilized blood-derived grafts is 4 × 10^6^/kg body weight. Therefore, we calculated the residual total cell number of potentially alloreactive TCRαβ^+^ T cells per 4 × 10^6^ CD34^+^ stem cells. TCRαβ^+^ T cell dose was reduced drastically by 4 log levels from > 100 × 10^6^ TCRαβ^+^ T cells per 4 × 10^6^ CD34^+^ stem cells in the starting material to a mean value of 20 × 10^3^ cells (range 1.8–35.8 × 10^3^ cells) in depleted products (Fig. 4H).

### Stability tests

**Fig. 5:**
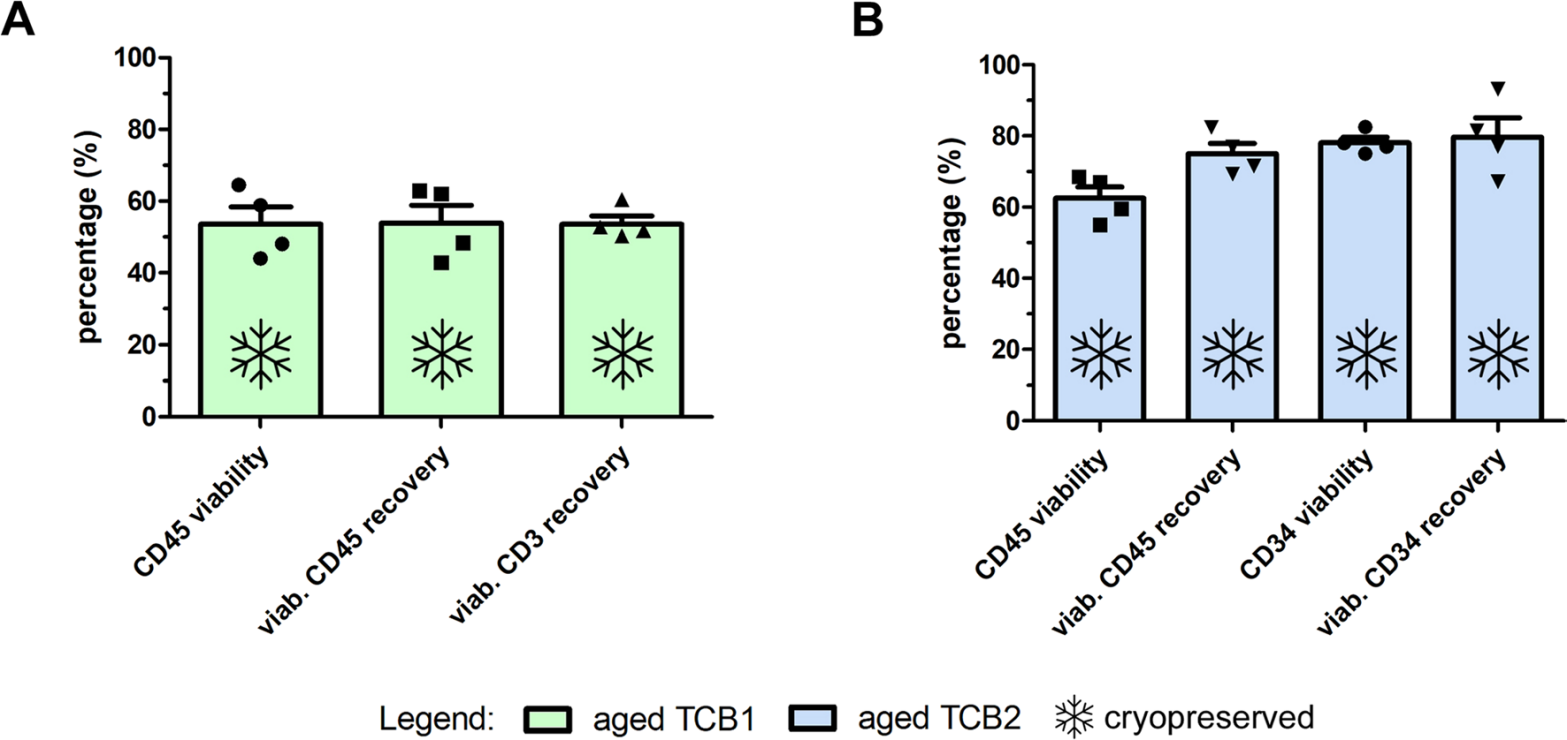
Post-thaw characterization of aged products cryopreserved at the end of their shelf life (72 h post-apheresis). The effect of a freeze–thaw procedure after storage at -180 °C for at least 1 month was analyzed on **A** aged CD45RA-depleted DLI product and **B** aged TCRαβ/CD19-depleted HSPC graft regarding the parameters CD45 viability, viable CD45 recovery, CD3 recovery or CD45 viability, viable CD45 recovery, CD34 viability and viable CD34 recovery, respectively. Individual runs depicted as dots as well as mean ± SEM are shown; n = 4 of each independent cryopreserved products of aged TCB1 and aged TCB2 from 4 freshly processed mobilized apheresis products per depletion process. Recovery values were calculated compared to the corresponding pre-cryopreserved fresh product

As either the apheresis product or the TCRαβ/CD19-depleted product aged, some loss of viable HSPCs was noted, as expected [27], but cell death was not noticeably different between the stored apheresis product and the stored TCRαβ/CD19-depleted product (Fig. 4B). Apheresis product that was processed 48 h after the end of collection was equally well depleted as a fresh product (Fig. 4C), but viability of CD34^+^ cells was significantly reduced compared to the freshly processed apheresis product, nevertheless still meeting the defined product specification of ≥ 70%. Mean overall yields of viable CD34^+^ cells, CD56^+^ NK cells and TCRγδ^+^ cells were 14.5%, 19.5% and 35% lower when older vs. fresh apheresis products were processed (p ≤ 0.05, < 0.001 and ns, respectively; Fig. 4E), whereas it was reduced by 5.3%, 4.3% and 16.9% for aged TCB2 product at the end of their shelf life (data not shown). Despite these differences, whether products were immediately processed or processed after 48 h, at the end of the fresh products’ shelf life of 72 h this did not result in dramatically different, or obviously inferior, product quality. Thus, both immediate and delayed processing is supported, although, because of higher viable CD34^+^ cell content of the “fresher” product, earliest possible manipulation and transfusion or cryopreservation would be preferred. We also cryopreserved 48-h aged cell products processed from fresh apheresis after CD45RA depletion and TCRαβ/CD19 depletion and thawed retained vials after a minimum of 1 month’s storage in LN2 vapor phase, and assessed several quality parameters pertinent to the respective product type, including CD45 viability, viable CD45 cell recovery as well as viable CD3 cell recovery, HSPC viability and recovery (Fig. 5). Predictably, total cell number estimates for all mature subsets for both product types were reduced after thawing compared to corresponding fresh products, reflecting a mean loss of viable WBCs of approximately 50% and 40% for CD45RA-depleted DLI products and TCRαβ/CD19-depleted HSPC grafts, respectively (Fig. 5A). Specifically, NK cells and granulocytes were significantly depleted after freeze-thawing (data not shown). Both, post-thaw CD45 and CD34 recovery in the TCRαβ/CD19-depleted graft resulted in robust mean values of 75% (range 69–82%) and 80% (range 77–93%) compared to their corresponding pre-cryopreserved product, respectively. Mean CD34^+^ cell viability was in the 80% range. Clonogenic growth was confirmed for 2/4 products tested in this fashion, the remaining two did not yield CFU-C (shown in Additional file 1: Figure S2). Although purely technical reasons cannot be definitively excluded, we interpret the data to indicate that cryopreservation of 72 h old cells, irrespective the time of their processing, may not be preferable.

Our data thus support, as for other immunomagnetically manipulated allogeneic stem cell products, a 72 h shelf life for not cryopreserved products and the possibility to release of both fresh and frozen HSPC and fresh and frozen DLI, depending on patient-individual needs.

## Discussion

We are presenting a protocol for generation of an allo-reactivity-reduced donor lymphocyte infusion (DLI) and an allo-reactivity-depleted HSPC product from the same mobilized apheresis harvest, both from one single-use tubing set. Both products have previously been generated using the same immunomagnetic reagents, albeit by two separate semi-manual immunomagnetic processes. The progress that our work represents is the realization of these manufacturing processes in a fully automatic, thus operator-independent process on CliniMACS Prodigy. Indeed a predecessor version of the TCRαβ/CD19 depletion technology was previously reported [10], using a different software and consumable, but in principle very similar reagents. Slightly inferior, but overall similar depletion of TCRαβ T cells and of B cells with similar recovery of HSPCs was achieved. At the time, the process could not be combined with the CD45RA-depletion module.

For both, the CD45RA-depleted mobilized DLIs [28–31] and the TCRαβ/CD19-depleted HSPC products [5, 31], clinical data supporting their effectiveness and safety, especially with regards to GVHD, was provided. Currently, TCRαβ-depleted grafts with or without B cell depletion, despite several reports of its good efficacy and low GvHD incidence, has not established itself as the standard for haplo-identical transplantation. Technical challenges of the earlier protocols as well as competition from the simple and clinically also quite satisfactory “PT/Cy” protocols may be the main reasons. The new process presented here can now support formal comparisons between unmanipulated PT/Cy-supported vs. TCRαβ/CD19-depleted HSPC.

At least initially, we foresee a fairly conventional approach to the use of the two medicinal products. The HSPC product will be infused after conditioning, DLI will be given prophylactically in very high-risk leukemia from week four, or therapeutically for re-emerging recipient chimerism or MRD. The dose of TCRαβ^+^ T cells/kg should typically not exceed 5 × 10^4^/kg in a haplo-identical constellation. At the discretion of the transplant physician, part of the HSPC product may be withheld and cryopreserved for potential future use if that dose was exceeded. If the resulting HSPC dose is deemed insufficient by the treating transplant physician, a second apheresis can be used for CD34+ cell enrichment, and the product used to supplement the HSPC dose. In case insufficient cells are collected in one apheresis and a second apheresis is performed, total WBC and target cell counts will determine whether apheresis products are processed sequentially, or pooled and processed together. Our stability data support processing of products as old as 48 h. For CD45RA-depleted DLI a starting dose of 1 × 10^5^/kg T cells was reported as a safe and effective starting dose [21]. Our recommendation would be to freeze at least five aliquots of increasing T cell content, 1 × 10^5^/kg, 3 × 10^5^/kg, 1 × 10^6^/kg, 3 × 10^6^/kg, 1 × 10^7^/kg; from a typical apheresis product, even considering the overall modest recovery of the CD45RA-depletion process, this should be feasible. Our suggested specification for both medicinal products to the regulatory agencies were added as Additional file 1: Table S2. Should the process fail technically, a second round of depletion can be considered; re-processing was a regular necessity during CD3/CD19-depletion [4]. However, the clinical role of both products remains to be fully defined.

The CD45RA depletion module on CliniMACS Prodigy used here is essentially the same that we previously reported, at that time including detailed functional and phenotypic characterization of the remaining T cells [15]. The salient difference between the current work and the paper by Müller et al. [15] is that here, mobilized as opposed to steady-state leukapheresis material was used, as in the pioneering study of CD45RA DLI [18]; there were also some minor differences in terms of the application, consumable and sample preparation. Although formal side-by-side comparison of starting materials was not pursued, the outcome data indicate that the process works approximately equally well with either cellular material.

The automatic process supports application of a younger HSPC product, since automatic manufacturing can be started immediately after the end of the apheresis and allowed to proceed overnight, whereas the manual process will typically not be started until the morning thereafter. The automatic process is highly transferable into other cell manufacturing labs and requires relatively less experience with cell therapy manufacturing. Our data demonstrate high robustness of the new automatic process with very satisfactory, consistent and clinically meaningful depletion efficiencies, efficient viable HSPC recovery and very high viability of all mature cell subsets. Where insufficient cells are collected in one apheresis and a second apheresis is performed, total WBC and target cell counts will determine whether apheresis products are processed sequentially, or pooled and processed together. Our stability data support processing of products as old as 48 h. Cryopreservation should be performed at the earliest opportunity, immediately after the end of the depletion process if possible. However, our data from cryopreservations performed at the end of the legally accepted shelf life for HSPC products in Germany, 72 h after the end of the apheresis, demonstrate that even at this late time point post-thaw quality was good. Confirmation of the targeted shelf life of 72 h for CD45RA-depleted DLI and TCRαβ/CD19-depleted HSPC product was one of the major goals of the stability exercise.

Considering the potential role of NK cells in tumor control and their virtual absence in post-thaw HSPC products, transplantation of fresh product should likely be given preference. However, recent data do not support superior outcomes with fresh vs. frozen unmanipulated HSPCs in a matched-donor setting [32] which would question a strong role of NK cells for tumor control. The possibility that the role of NK cells in allo-transplantation is only unmasked when T cells are depleted may be entertained.

Our work does not help clarifying the future role of TCRαβ/CD19-depleted grafts as haplo-identical transplant, as cheaper alternatives exist [33, 34]. All have proven their clinical effectiveness, but side-by-side comparisons including functional immune reconstitution and long-term toxicity remain to be studied. Definitive studies as to the clinical role of CD45RA and TCRαβ/CD19 depletion for GVHD-reduced allo-transplantation are now enabled by the work presented in this manuscript, since the fully automatic sequential CD45RA and TCRαβ/CD19 depletion process enables easy dissemination of this technology. The robustness of the method opens options for de-centralized manufacturing, due to the high degree of automation of both manufacturing and the most sensitive quality control assays, i.e., flow cytometry analyses. More broadly, the data underscore the impression of high versatility of the CliniMACS Prodigy platform.

### Supplementary Information

**Additional file 1: Table S1.** Summary table of process details of the ten CD45RA (a) and TCRαβ/CD19 depletion runs (b). **Table S2.** Our suggested release criteria for both medicinal products. **Figure S1.** Description of the components and preparation steps of the CliniMACS Prodigy TS 320 setup. **Figure S2.** Post-thaw characterization of fresh products generated from aged leukapheresis product cryopreserved at the end of their shelf-life (72 h post-apheresis).

## Data Availability

All data relevant to the study are included in this published article. The datasets generated and analyzed during the current study are not publicly available due to the confidentiality agreement with Miltenyi Biotec B.V. & Co. KG but are available from the corresponding author on reasonable request.
