# Supplementary material for: Automatic generation of alloreactivity-reduced donor lymphocytes and hematopoietic stem cells from the same mobilized apheresis product
Source: J Transl Med. 2023 Nov 25;21:849. doi: 10.1186/s12967-023-04738-8 (PMC10675913; doi:10.1186/s12967-023-04738-8)

Additional information

Table S1. Summary table of process details of the ten CD45RA (a) and TCRαβ/CD19 depletion runs (b).

a)

Run No.	[1]Vol. ml	Process scale	Mob LP (starting material)							CD45RA-depleted DLI (TCB1), fresh									
			[2]Vol.	WBC	CD3	CD45RA	CD20	CD34	CD56	[2]Vol.	[3]Vol	Log depletion		WBC	CD3	CD45RA	CD20	CD34	CD56
			ml	10^6	10^6	10^6	10^6	10^6	10^6	ml	ml	CD45RA	CD19	10^6	10^6	10^6	10^6	10^6	10^6
1	179	NS	17	3830	1220	1060	210	48.5	396	17	124	4.4	3.8	628	57.6	0.042	0.0033	13.5	10
2	258	LS	14	3590	1170	1070	210	24	242	14	121	4.3	4.24	797	41	0.0054	0.0012	5.04	10.06
3	82	LS	17	3770	1470	1160	91	41.1	282	17	128	4.11	3.48	717	62.7	0.0911	0.003	16	5.56
4[a]	84	LS	17	3550	1330	1330	103	41.5	273	17	127	4.07	2.61	454	47.8	0.113	0.255	10.7	1.03
5	134	LS	15	3620	1200	940	549	54.8	231	15	112	4.19	4.39	502	96.6	0.0061	0.0023	19.4	2.86
6[a]	134	LS	15	2000	901	407	501	28.8	96.5	15	112	3.58	3.89	288	50.8	0.107	0.0064	10.1	1.43
7	110	NS	27	4870	2080	947	187	50.8	600	27	114	4.66	4.69	864	205	0.0209	0	18.1	11.7
8[a]	109	NS	27	4080	1700	1250	170	41.4	379	27	116	4.26	3.96	761	184	0.0068	0	18.7	3.63
9	146	NS	20	4690	1430	782	376	58	430	20	123	4.13	3.96	951	157	0.0058	0.0041	27.3	3.23
10[a]	148	NS	20	3870	1080	765	353	60.5	408	20	114	3.96	3.89	910	141	0.0084	0.0458	29.7	4.37
Mean	138.4	/	18.90	3787	1358	971.1	275	44,94	333.8	18.90	119.1	4.17	3.89	687	104.4	0.0407	0.0321	16.85	5.39
Min.	82	/	14.00	2000	901	407	91	24	96.50	14	112	3.58	2.61	288	41	0.0054	0	5.04	1.03
Max.	258	/	27.00	4870	2080	1330	549	60,50	600	27	128	4.66	4.69	951	205	0.1130	0.255	29.7	11.7

b)

Run No.	[1]Vol. ml	Process scale	Mob LP (starting material)							TCRαβ/CD19-depleted HSPCs (TCB2), fresh									
			[2]Vol.	WBC	CD3	TCRαβ	CD20	CD34	CD56	[2]Vol.	[3]Vol	Log depletion		WBC	CD3	TCRαβ	CD20	CD34	CD56
			ml	10^6	10^6	10^6	10^6	10^6	10^6	ml	ml	TCRαβ	CD19	10^6	10^6	10^6	10^6	10^6	10^6
1	179	NS	162	36500	11600	14300	2000	367	3770	162	242	3.98	4.25	17800	748	1.49	0.114	290	3060
2	258	LS	244	62500	20400	22400	3660	459	4220	244	311	3.99	4.16	34300	863	2.31	0.25	283	3450
3	82	LS	65	14300	5600	5810	346	168	1070	65	274	4.99	3.43	5570	68.6	0.060	0.128	135	545
4[a]	84	LS	67	14000	5230	6170	405	164	1070	67	271	4.29	3.35	4950	53.5	0.319	0.182	85	405
5	134	LS	119	28700	9520	10500	4350	484	1830	119	261	4.18	4.3	10600	312	0.68	0.219	365	1330
6[a]	134	LS	119	15900	7150	4490	3970	368	766	119	260	3.60	4	7540	167	1.61	0.393	188	617
7	110	NS	83	17900	6390	6190	574	156	1840	83	258	4.53	4.12	5810	126	0.183	0.0432	123	1150
8[a]	109	NS	82	12400	5160	5700	518	126	1150	82	269	3.8	3.53	4380	79	0.904	0.153	101	530
9	146	NS	126	29500	9030	8480	2370	365	2710	126	218	3.55	4.5	14400	421	2.39	0.0005	381	2110
10[a]	148	NS	128	24700	6920	7590	2260	387	2610	128	219	3.78	3.89	11200	237	1.27	0.293	403	1090
Mean	138.4	/	119.5	25640	8700	9163	2045	304.4	2104	119.5	258,3	4.07	3.95	11655	307.5	1.122	0.1776	235.4	1429
Min.	82	/	65	12400	5160	4490	346	126	766	65	218	3.44	3.35	4380	53.50	0.060	0.0005	85	405
Max.	258	/	244	62500	20400	22400	4350	484	4220	244	311	4.99	4.5	34300	863	2.390	0.3930	403	3450

[a]Mobilized leukapheresis (mob LP) stored for 48 hours at 2-6 °C was used as the starting material for the depletion process.
[1]Vol: Total volume of mobilized leukapheresis product.
[2]Vol: Process volume used for depletion process.
[3]Vol: End volume of target product provided in Target Cell Bag.

Table S2: Our suggested release criteria for both medicinal products

Test parameter	Method/ Assay	Acceptance criteria
CD45RA-depleted DLI		
Sterility	BacT/ALERT	Negative or no growth
Viability $CD45^+$ cells	flow cytometry (7-AAD)	≥ 90 % pre-freeze<br>≥ 50 % post-thaw
Cell Count (WBC)	Sysmex and/or flow cytometry	Determined and declared
$CD3^+$ cells	flow cytometry	Determined and declared<br>Suggested doses: $1x10^5$/kg, $3x10^5$/kg, $1x10^6$/kg, $3x10^6$/kg, $1x10^7$/kg
$CD45RA^+$ T cells	flow cytometry	Determined and declared
$CD20^+$ B cells	flow cytometry	Determined and declared
IDMs	NAT, serology	Negative for hematogenically transmissible agents
TCRαβ/CD19-depleted HSPCs		
Sterility	BacT/ALERT	Negative or no growth
Viability $CD45^+$ cells	flow cytometry (7-AAD)	≥ 90 % pre-freeze
Cell Count (WBC)	Sysmex and/or flow cytometry	Determined and declared
$CD34^+$ cells	flow cytometry	≥ 4 x 10^6 / kg (target, >90% of all products)
Viability $CD34^+CD45^+$ cells	flow cytometry	≥ 90 % pre-freeze<br>≥ 70 % post-thaw
$CD3^+$ T cells	flow cytometry	Determined and declared
$TCRαβ^+$ T cells	flow cytometry	Determined and declared
$CD20^+$ B cells	flow cytometry	Determined and declared
IDMs	NAT, serology	Negative for hematogenically transmissible agents

Figure S1: **Description of the components and preparation steps of the CliniMACS Prodigy TS 320 setup.**

The LP-TCRab-19-45RA System allows fully automated sequential depletion of CD45RA and of TCRαβ±CD19 expressing cells on the CliniMACS Prodigy device, using the same tubing set. Only few preparation steps are required. Ports are loaded as indicated in the graphic, using sterile filter-equipped spike or luer ports or sterile tube welding.

A first optional process deposits CD45RA depleted product in Target Cell Bag 1 (TCB1). Before bulk depletion in the second process (TCRαβ/CD19 or TCRαβ depletion), the TCRαβ/CD19-labelled cell product is applied to the Filtration Bag to remove potential cell aggregates. The Reapplication Bag serves for temporary storage of bulk depleted product before concentration in the CentriCult unit. The TCRαβ/CD19-depleted product is suspended in infusion solution, then deposited into Target Cell Bag 2 (TCB2). The Non-Target Cell Bag contains the column eluate with labelled cells.

Copyright © 2023 Miltenyi Biotec B.V. & Co. KG. All rights reserved.

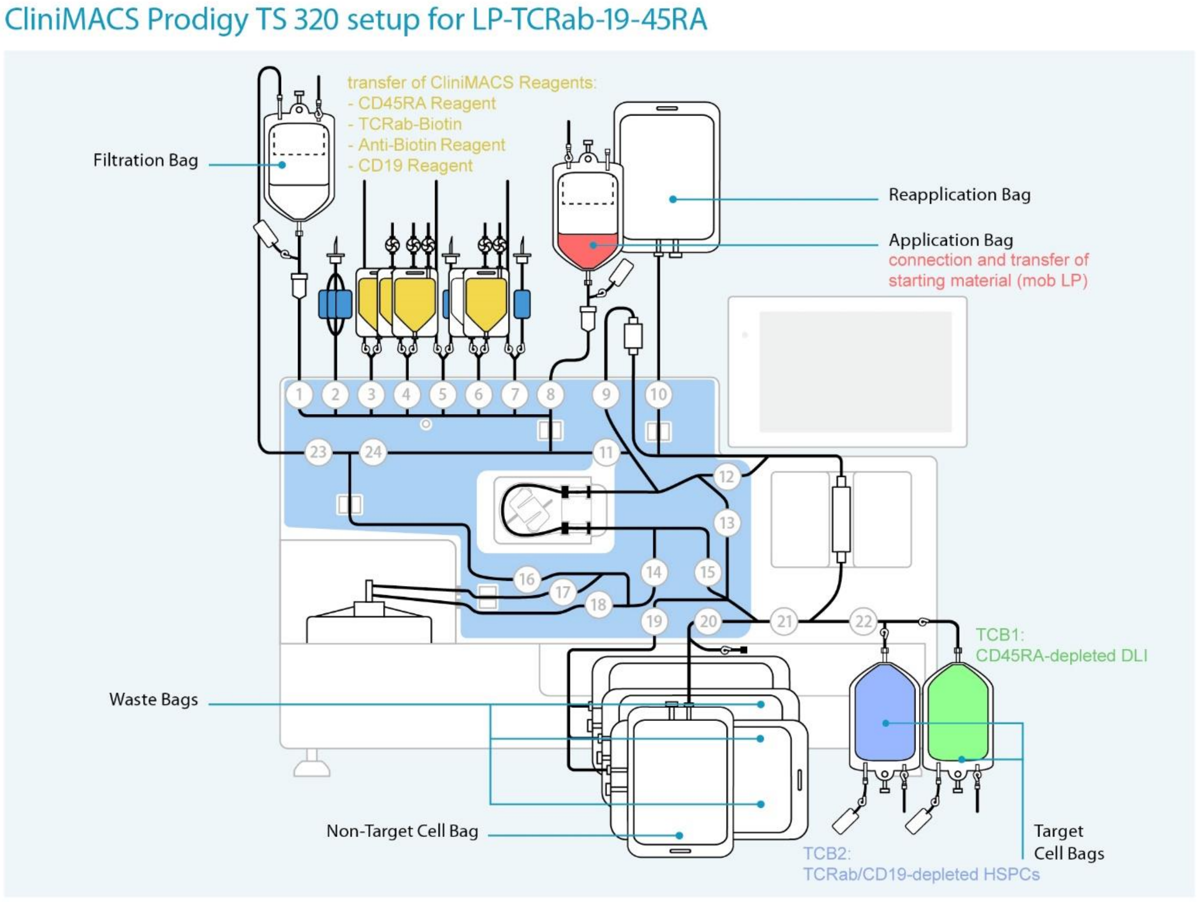

Figure S2: **Post-thaw characterization of fresh products generated from aged leukapheresis product cryopreserved at the end of their shelf-life (72 hours post-apheresis).**

The effect of a freeze-thaw procedure after storage at -180 °C for at least 1 month was analyzed on **(A)** fresh CD45RA-depleted DLI product and **(B)** fresh TCRαβ/CD19-depleted HSPC graft after processing of aged mob LP regarding the parameters CD45 viability, viable CD45 recovery or CD45 viability, viable CD45 recovery, CD34 viability and viable CD34 recovery, respectively. Individual runs depicted as dots as well as mean ± SEM are shown; n = 4 of each independent freshly cryopreserved product of TCB1 and TCB2 processed from 4 aged mobilized apheresis products per depletion process. Recovery values were calculated compared to the corresponding pre-cryopreserved fresh product. **(C)** CFU-C assays were performed after a freeze-thaw procedure on aged TCB2 derived from fresh mob LP and on fresh TCB2 derived from aged mob LP, respectively.

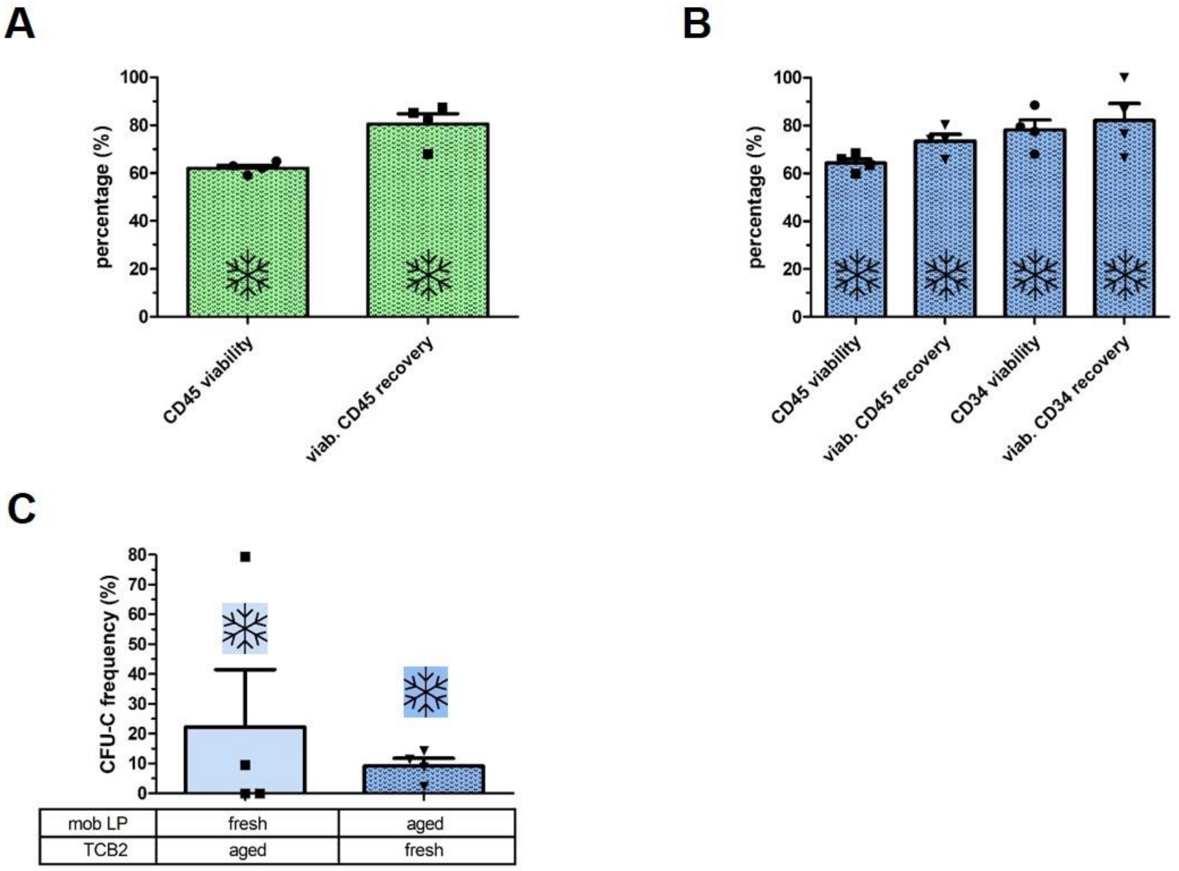

Supplement: Supplementary file 1 — Additional file 1: Table S1. Summary table of process details of the ten CD45RA (a) and TCRαβ/CD19 depletion runs (b). Table S2. Our suggested release criteria for both medicinal products. Figure S1. Description of the components and preparation steps of the CliniMACS Prodigy TS 320 setup. Figure S2. Post-thaw characterization of fresh products generated from aged leukapheresis product cryopreserved at the end of their shelf-life (72 h post-apheresis). [file 12967_2023_4738_MOESM1_ESM.pdf]
